# Complement Receptor 3 Regulates Microglial Exosome Release and Related Neurotoxicity via NADPH Oxidase in Neuroinflammation Associated with Parkinson’s Disease

**DOI:** 10.3390/antiox14080963

**Published:** 2025-08-05

**Authors:** Yu Ma, Xiaomeng Zhang, Jiaqi Xu, Runnan Luo, Sheng Li, Hong Su, Qingshan Wang, Liyan Hou

**Affiliations:** 1 Department of Health Toxicology, School of Public Health, Dalian Medical University, Dalian 116044, China; may11@dmu.edu.cn (Y.M.);; 2 National-Local Joint Engineering Research Center for Drug-Research and Development (R&D) of Neurodegenerative Diseases, Dalian Medical University, Dalian 116044, China; zhangxm2853@163.com (X.Z.);; 3 School of Health-Preservation and Wellness, Dalian Medical University, Dalian 116044, China; 4 Dalian Medical University Library, Dalian Medical University, No. 9 W. Lvshun, South Road, Dalian 116044, China

**Keywords:** complement receptor 3 (CR3), NADPH oxidase (NOX2), exosome, syntenin-1, neuroinflammation, Parkinson’s disease (PD)

## Abstract

Microglia-mediated chronic neuroinflammation is a common pathological feature of Parkinson’s disease (PD). Strong evidence suggests that activated microglia can lesion neurons by releasing exosomes. However, the mechanisms of exosome release from activated microglia remain unclear. We recently revealed a key role of complement receptor 3 (CR3) in regulating microglial activation in the process of progressive neurodegeneration. This study aimed to investigate whether CR3 can regulate exosome release from activated microglia, as well as the underlying mechanisms. We found that LPS, an inducer of microglial M1 activation, induced exosome release from activated microglia. Inhibition of exosome synthesis suppressed LPS-induced microglial activation, gene expression of proinflammatory factors, and related neurotoxicity. Silencing or knocking out CR3 attenuated LPS-induced exosome release in microglia. NADPH oxidase (NOX2) was further identified as a downstream signal of CR3, mediating microglial exosome release and related neurotoxicity. CR3 silencing blocked LPS-induced NOX2 activation and superoxide production through inhibition of p47^phox^ phosphorylation and membrane translocation. Moreover, NOX2 activation elicited by PMA or supplementation of H_2_O_2_ recovered exosome release from CR3-silenced microglia. Subsequently, we demonstrated that the CR3-NOX2 axis regulates syntenin-1 to control microglial exosome release. Finally, we observed that the expression of CR3 was increased in the brain of LPS-treated mice, and genetic ablation of CR3 significantly reduced LPS-induced NOX2 activation, microglial M1 polarization, and exosome production in mice. Overall, our findings revealed a critical role of the CR3-NOX2 axis in controlling microglial exosome release and related neurotoxicity through syntenin-1, providing a novel target for the development of a therapeutic strategy for neuroinflammation-mediated neurodegeneration.

## 1. Introduction

The first detailed description of Parkinson’s disease (PD) was made by humans two centuries ago, but the concept of the disease is still evolving [1]. PD is a complex neurological system disease, at the core of which is neurodegeneration. It is characterized by the early and significant death of dopaminergic neurons in the substantia nigra pars compacta (SNpc) [2]. This leads to the occurrence of typical PD symptoms, including bradykinesia, muscular rigidity, resting tremor, and gait disturbance [3].

The pathogenesis of PD is very complex, including protein homeostasis and damage to cells that regulate homeostasis, aberrations and abnormalities in synaptic structure and function, and mitochondrial dysfunction [4]. In recent years, the role of immune and neuroinflammation regulated by microglia in the development and progression of PD has received increasing attention. As an immune barrier of the central nervous system (CNS), microglia can engulf cell debris produced during host defense and pathogens. However, under continuous external stimulation, microglia can rapidly activate and secrete inflammatory factors, leading to neuroinflammation and further accelerating the course of PD [5]. Microglia exist in two polarized activation modes: M1 and M2. M1 polarization is known as classical activation, which can produce pro-inflammatory factors and release neurotoxicity. M2 polarization is known as selective activation, which has anti-inflammatory effects and repairs nerve damage. Modulating the phenotype of microglial, switching from the pro-inflammatory M1 polarization to the anti-inflammatory M2 polarization, provides a new approach for the treatment of PD. However, the mechanism by which the polarization of microglia occurs is still unclear.

Complement receptor 3 (CR3), also known as CD11b/CD18, is highly expressed in innate immune cells such as microglia [6]. According to reports, CR3 in microglia can recognize a variety of signaling molecules to mediate neurodegenerative lesions [7]. In microglia, CR3 can trigger an inflammatory response and the production of free radicals by interacting with aggregated α-synuclein (α-syn), thereby exacerbating neurotoxicity [8]. The absence of CR3 can lead to the inhibition of microglial activation in PD model mice, suggesting that CR3 plays an important role in the progression of PD. NADPH oxidase (NOX2), a superoxide enzyme produced by microglia, can be regulated by CR3 and plays a role in the pathogenesis of PD [9]. Our previous evidence confirmed that CR3 can respond to α-syn, a major component of Lewy bodies in PD, and thereby regulate NOX2 to promote the progression of PD [8]. Although the mechanism by which CR3 regulates the role played by NOX2 in PD has been extensively reported, the main mechanism is the generation of large amounts of reactive oxygen species (ROS) via NOX2 in microglia, thereby causing oxidative stress and ultimately leading to neuroinflammation as well as the onset of neurodegeneration [10].

Exosomes are small extracellular vesicles, which have now been shown to be involved in a variety of pathological processes, such as cancer, cell senescence, and PD [11]. Recently, it has been shown that microglia activated to the M1 phenotype can efficiently secrete exosomes as part of their antigen presentation and release of inflammatory factors [12]. Our study also revealed that NOX2 activation promotes microglia M1 polarization and exosome production and releases large amounts of inflammatory factors, resulting in dopaminergic neurodegeneration [13]. Whether CR3 can regulate microglia exosome release through NOX2 and the exact mechanism is unknown. The multifunctional protein Syntenin-1, a key protein in exosome synthesis and release, has been shown to mediate the FAK-Src signaling pathway through interaction with integrins to regulate cell adhesion and growth [14]. However, it has not been reported whether it is regulated by the CR3-NOX2 axis in microglia and is involved in the release of exosomes, leading to neurodegeneration.

In this article, we mainly aim to investigate whether the CR3-NOX2 axis can cause neuroinflammation and neurodegeneration by regulating the release of microglial exosomes, and strive to reveal the mechanism by which the CR3-NOX2 axis regulates the release of microglial exosomes. It is hoped to provide new laboratory evidence for the pathogenesis of PD.

## 2. Materials and Methods

### 2.1. Materials

LPS (99%), 3-(4,5-Dimethylthiazol-2yl-)-2,5-diphenyltetrazolium bromide (MTT) (>98%), and dimethyl sulfoxide (DMSO) (>99%) were purchased from Beyotime Institute of Biotechnology (Shanghai, China). NOX2 activator phorbol myristate acetate (PMA) (99.8%) and exosome synthesis/release inhibitor GW4869 (98.86%) were obtained from MedChemExpress (Shanghai, China). H_2_O_2_ (30%) was acquired from Tianjin Kemiou Chemical Reagent Co., Ltd. (Tianjin, China). Dulbecco’s Modified Eagle Medium (DMEM), serum-free medium, Fetal Bovine Serum (FBS), Sodium Pyruvate, and L-Glutamine were purchased from Gibco, Thermo Fisher Scientific (Waltham, MA, USA). Penicillin/streptomycin (100×) was acquired from Titan Scientific (Shanghai, China).

### 2.2. Cell Culture

BV2 microglia were cultured in DMEM containing 10% FBS, 1% penicillin/streptomycin, 1% sodium pyruvate, and L-glutamine. Human neuroblastoma (SH-SY5Y) cells were cultured in DMEM containing 10% FBS and 1% penicillin/streptomycin. The cells were cultured in a cell incubator at 37 °C with 5% CO_2_. Both cells were purchased from Wuhan Pricella Biotechnology Co., Ltd. (Wuhan, China).

### 2.3. Animals

Wild-type (WT) adult male C57BL/6 mice and CR3^−/−^ mice (a total of 40 animals) obtained from Liaoning Changsheng Biotechnology Co., Ltd. (Liaoning Sheng, China) were randomly divided into control and LPS treatment groups using the random number table method. LPS-treated group: Mice were given an intraperitoneal injection (i.p.) of 0.5 mg/kg LPS for 24 h (n = 10 per group). Control mice were treated with an equivalent amount of PBS (n = 10 per group). LPS has been widely used to induce neuroinflammation in mice, and the number of animals in each group was determined based on reports [15]. The experimental animals were allowed to drink and eat freely. Ms. Xiaomeng Zhang was aware of the grouping of the animals and handled them. If during the experiment a mouse showed signs of extreme pain or distress or any noticeable change in behavior, it was removed from the experiment and euthanized. We provided experimental protocols, and the ethics of this experiment were approved by the Dalian Medical University Ethics Committee (AEE21016). All animal experiments were performed in accordance with relevant regulations.

### 2.4. Extraction and Identification of Exosomes

Exosomes were extracted using exosome isolation reagents (4478359 and 4478360, Invitrogen™, Waltham, MA, USA). For BV2 cells, cells were inoculated in 10 cm dishes and cultured in serum-free medium. The cell supernatant was collected at the end of the treatment of each group and centrifuged at 2000× *g* for 30 min to remove cell debris. The supernatant was transferred to a new centrifuge tube, and one-half volume of isolation reagent was added, mixed well, and left to stand overnight at 4 °C. After incubation, the samples were centrifuged at 10,000× *g* for 1 h at 4 °C. The precipitate contained the exosomes. For mice, the serum in each treatment group was centrifuged at 2000× *g* for 30 min to remove cell debris, the supernatant was transferred to a new centrifuge tube, and one-fifth of the volume of the separation reagent was added. After mixing thoroughly, the sample was incubated at 4 °C for 30 min. Then, the sample was centrifuged at 10,000× *g* for 10 min. The particles at the bottom of the tube were exosomes.

Exosomes were observed using TEM to identify their morphology, and Western blot was used to detect the expression of exosome marker proteins TSG101 and CD63, as in other studies [16]. The contents of exosomes were detected using the BCA protein quantification method [17].

### 2.5. Western Blot Analysis

After each group of cells or mice was treated, RIPA lysis buffer (containing protease and phosphatase inhibitors) was used to lyse the samples on ice for 30 min. Protein samples were quantified using the BCA protein assay and then subjected to electrophoresis on sodium dodecyl sulfate-polyacrylamide gel (SDS-PAGE). The protein strips were transferred to a polyvinylidene difluoride (PVDF) membrane and blocked with skimmed milk for 2 h. The membrane was washed three times with TBST and then incubated with the different primary antibodies overnight at 4 °C. Secondary antibodies were added, and the membrane was incubated for 2 h, after which proteins were visualized using enhanced chemiluminescence (ECL). The results were analyzed using the Image J software (Version:1.52a).

For the extraction of cell membrane and cytoplasmic proteins, the Membrane and Cytosol Protein Extraction kit (Beyotime, Shanghai, China) was used. The rest was processed as described above.

The following primary antibodies were used in this study: phospho-p47^phox^ (Ser345), p47^phox^ (ab308256), TSG101, and CD63 were purchased from Abcam (Cambridge, UK). CR3 (A24120), gp91^phox^ (A19701), and Tyrosine Hydroxylase (TH) (A0028) were obtained from ABclonal Biotechnology Co., Ltd. (Wuhan, China). Syntenin-1 (22399-1-AP) and GAPDH (10494-1-AP) were acquired from Proteintech (Wuhan, China). HRP-labeled Goat Anti-Rabbit IgG (H + L), which was acquired from Beyotime Biotechnology (Nantong, China), was used as the secondary antibody.

### 2.6. MTT Assay

BV2 or SH-SY5Y cells were seeded at 8000 cells per well in a 96-well plate and treated for 20 h. MTT was added to each well, and the cells were incubated for a further 4 h. After discarding the medium, 200 μL of DMSO was added to each well, and the absorbance was measured at a wavelength of 492 nm after complete dissolution of the formazan. cell viability (%) = (A treatment/A control) × 100%.

### 2.7. Quantitative Real Time Polymerase Chain Reaction (qRT-PCR)

TRIzol^®^ reagent (Invitrogen, Waltham, MA, USA) was used to extract total RNA. HiScript^®^ II Q RT SuperMix for qPCR (+gDNA wiper) (Vazyme, Nanjing, China) was used to synthesize cDNA, and ChamQ™ Universal SYBR^®^ qPCR Master Mix (Vazyme, Nanjing, China) was used to perform qRT-PCR according to the manufacturer’s protocol. The Ct (2^−ΔΔCt^) method was used for data analysis.

The sequences of the primers used in this study are as follows: 5′-CTGGTGTGTGACGTTCCCATTA-3′ (F), 5′-CCGACAGCACGAGGCTTT-3′ (R) for *IL-1β*, 5′-CTGCCCCCCTGCTCACTC-3′ (F), 5′- TGGGAGGGGTCGTAATGTCC-3′ (R) for *iNOS*, 5′-GACCCTCACACTCAGATCATCTTCT-3′ (F), 5′-CCTCCACTTGGTGGTTTGCT-3′ (R) for *TNF-α*, 5′-GAACACGGCAGTGGCTTTAAC-3′ (F), 5′-TGCTTAGCTCTGTCTGCTTTGC-3′ (R) for *Arg1*, 5′-AAGGAAGGTTGGCATTTGT-3′ (F), 5′-CCTTTCAATCCTATGCAAGC-3′ (R) for *CD206*, 5′-TTCAACGGCACAGTCAAGGC-3′ (F), 5′-GACTCCACGACATACTCAGCACC-3′ (R) for *GAPDH*.

### 2.8. Cell Transfection

The transfection reagent GP-transfect-Mate (GenePharma, Shanghai, China) was used according to the instructions. The operation was performed when the BV2 cell density reached about 50%. After 6 h of transfection, the serum-free medium was replaced with normal medium, and the cells were incubated for a further 18 h.

### 2.9. Superoxide Detection

The Superoxide Assay Kit from Beyotime Biotechnology (Shanghai, China) was used for superoxide detection in BV2 microglia treated with LPS, with or without CR3-siRNA. BV2 microglia were inoculated in 96-well plates at 8000 cells per well, and when the cell density reached about 80%, the medium was discarded and the cells were washed once with PBS. A 200 μL volume of working solution was added, and the mixture incubated at 37 °C for 3 min. Different treatments were added, and the mixtures incubated at 37 °C for 4 h. The absorbance was then measured at 450 nm. The superoxide content in each group of cells was calculated according to the instructions.

### 2.10. Immunocytochemistry

After treating the cell groups, the cells were washed with PBS, incubated with H_2_O_2_ for 10 min, and then blocked with 0.25% Triton/PBS containing 4% goat serum for 2 h. The cells were then analyzed using anti-TH (1:1000) antibody. Immunostaining was performed using DAB.

### 2.11. Statistical Analysis

The results are presented as mean ± standard deviation (SD). GraphPad Prism 8.0 was used for graphing and statistical analysis. The *t*-test was used to analyze the difference between two samples, and one-way ANOVA, followed by SNK-q test, was used to analyze the difference between multiple samples. All experiments were repeated at least three times, and *p*-values less than 0.05 were considered to indicate statistically significant differences.

## 3. Results

### 3.1. LPS-Induced M1 Microglia Damages Neurons Through Release of Exosomes

**Figure 1:**
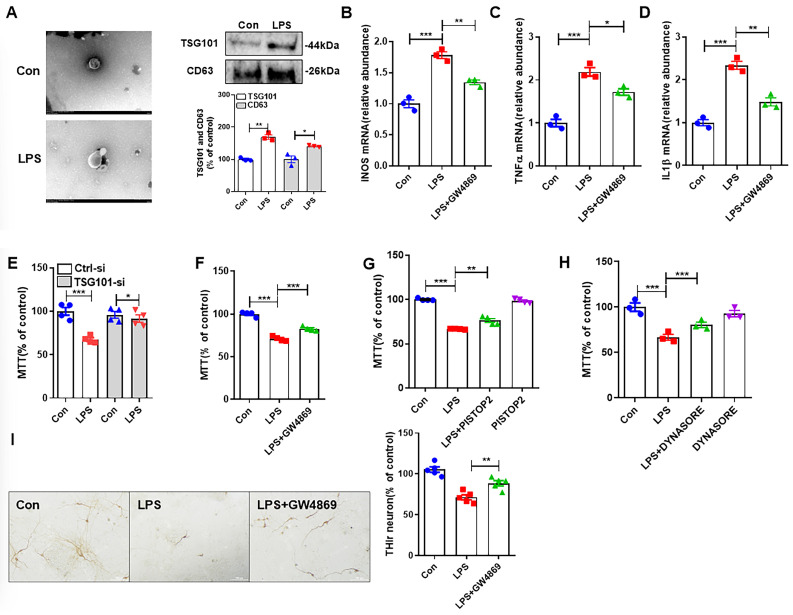
LPS-induced M1 microglia damage neurons through the release of exosomes. (**A**) TEM identified exosome formation, and Western Blot detected the exosome marker proteins TSG101 and CD63. (**B**–**D**) qRT-PCR was performed to detect iNOS, TNF-α, and IL-1β mRNA expression with or without GW4869. (**E**–**H**) Detection of cell viability by MTT assay with or without TSG101-si, GW4869, PISTOP2, and DYNASORE. (**I**) TH immunostaining in LPS-intoxicated primary midbrain cultures with or without GW4869. Scale bar = 100 μm. * *p* < 0.05, ** *p* < 0.01, *** *p* < 0.001. n = 3–6.

To investigate whether microglial activation-mediated neurotoxicity was caused by exosome release, TEM was initially used to characterize the formation of exosomes in BV2 cells, and a hemispherical exosome structure with one concave side was observed in both vehicle and LPS-treated cells (Figure 1A). Compared with the control group, the expression of exosome-related proteins TSG101 and CD63 was also significantly elevated in BV2 cells after 24 h of LPS treatment (Figure 1A), indicating elevated exosome release from activated microglia. Figure 1B–D demonstrate the effects of adding LPS treatment for 24 h on M1 polarization of BV2 cells with or without using 10 μM exosome synthesis inhibitor GW4869. The mRNA expression of iNOS, TNF-α, and IL-1β was significantly decreased when exosome synthesis was inhibited compared with the LPS-treated group.

To further clarify the role of exosomes in mediating microglial activation-related neuronal injury, we knocked down TSG101 in BV2 cells. SH-SY5Y cells were treated for 24 h with conditioned medium prepared from the TSG101 knockdown BV2 cells with or without LPS treatment. It was found that LPS-induced reduction of SH-SY5Y cell viability was alleviated by TSG101 knockdown (Figure 1E). For the same purpose, we treated BV2 cells with 10 μM exosome synthesis inhibitor GW4869 and found that inhibition of microglial exosome synthesis by GW4869 also attenuated LPS-induced reduction of cell viability in SH-SY5Y cells. Exosomes released from activated microglia must be taken up by neurons to damage them. The SH-SY5Y cells were therefore treated with clathrin inhibitor PISTOP2 (20 μM) or dynamin inhibitor DYNASORE (10 μM) to inhibit endocytosis and were then intoxicated with conditioned medium prepared from LPS-treated BV2 cells. PISTOP2 and DYNASORE treatment effectively alleviated the decrease in cell viability of SH-SY5Y cells caused by LPS (Figure 1F–H). These results confirmed that the presence or absence of CR3 modulates M1 polarization, NOX2 activation, and exosome release from microglia in LPS-treated mice.

Primary midbrain neuron-glia culture treated with LPS is widely used in in vitro PD models. LPS treatment elicited a neuroinflammation-dependent loss of dopaminergic neurons by showing a reduced number of TH-immunoreactive cells (Figure 1I). Inhibition of exosome synthesis also reduced LPS-induced loss of dopaminergic neurons in primary cultures (Figure 1I). Based on these results, we clarified that LPS can cause neuronal damage by inducing M1 microglia through the release of exosomes.

**Figure 2:**
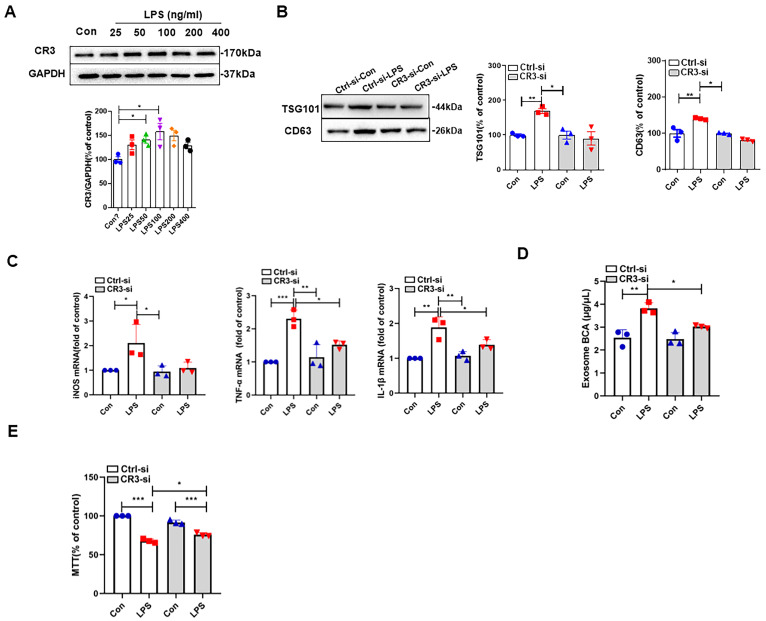
CR3 mediates microglial M1 polarization, NOX2 activation, and exosome release in the brain of LPS-treated mice. (**A**) CR3 protein expression after treatment of BV2 cells with different concentrations of LPS. (**B**) TSG101 and CD63 protein expression after LPS treatment of BV2 cells with or without CR3-si. (**C**) qRT-PCR was performed to detect iNOS, TNF-α, and IL-1β mRNA expression with or without CR3-si. (**D**) BCA protein quantification for the detection of exosome release with or without CR3-si. (**E**) Detection of cell viability by MTT assay with or without CR3-si. * *p* < 0.05, ** *p* < 0.01, *** *p* < 0.001. n = 3.

In this section, we will explore whether CR3 was involved in LP3-induced exosome release from M1 microglia and related neurotoxicity. After treating BV2 cells with different concentrations of LPS for 24 h, it was found that 100 ng/mL LPS caused the highest expression of CR3; thus, this concentration was used for treatment thereafter (Figure 2A). When we knocked down CR3, we found that LPS induced the expression of the exosome marker protein TSG101, while CD63 expression was significantly alleviated (Figure 2B). CR3 deletion significantly attenuated the LPS-induced elevation of microglia M1 polarization markers iNOS, TNF-α, and IL-1β mRNA expression (Figure 2C). BCA protein quantification demonstrated that the absence of CR3 significantly reduced LPS-induced exosome release from BV2 cells (Figure 2D). After treating BV2 cells with LPS for 24 h, the cell supernatant (conditioned medium) was collected and administered to SH-SY5Y cells for 24 h. SH-SY5Y cell viability was detected using MTT. The results suggested that the absence of CR3 could significantly reduce the release of exosomes from BV2 cells, which in turn alleviated LPS-induced neurotoxicity (Figure 2E). These results clarified the involvement of CR3 in LPS-induced release of exosomes from M1 microglia and the neurotoxicity caused.

### 3.2. CR3 Mediates M1 Microglia Exosome Release via NOX2

**Figure 3:**
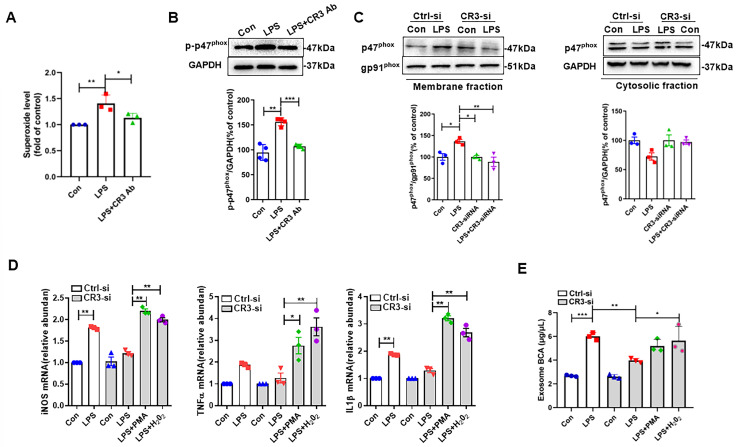
CR3 mediates M1 microglia exosome release via NOX2. (**A**) Superoxide level with or without CR3-Ab. (**B**) Protein p47^phox^ was detected using Western blot with or without CR3-Ab. (**C**) LPS-induced p47^phox^ membrane translocation was detected using Western blot with or without CR3-si. (**D**) mRNA expression of iNOS, TNF-α, and IL-1β detected using qRT-PCR with or without CR3-si, PMA, and H_2_O_2_. (**E**) BCA protein quantification for the detection of exosome release with or without CR3-si, PMA, and H_2_O_2_. * *p* < 0.05, ** *p* < 0.01, *** *p* < 0.001. n = 3–4.

As a downstream effector molecule of CR3, the role of NOX2 in CR3 regulation of microglia M1 polarization has been reported in our previous studies [13]. However, whether CR3 can regulate the release of exosomes from M1 microglia through NOX2 is unknown. The results in Figure 3A showed that LPS could induce increased levels of superoxide in BV2 cells, while superoxide production was decreased when the CR3 antibody was added. Meanwhile, the expression of p-p47^phox^ protein showed the same change (Figure 3B). For the expression of p47^phox^ protein in the cell membrane and cytosol, it was confirmed that LPS could promote the expression of p47^phox^ in the BV2 cell membrane and decrease its expression in the cytosol, suggesting that membrane translocation of p47^phox^ occurs. However, the absence of CR3 prevented LPS-induced p47^phox^ membrane translocation (Figure 3C), demonstrating that CR3 mediates LPS-induced NOX2 activation through p47phox phosphorylation and subsequent membrane translocation. Figure 3D demonstrated the changes in microglia M1 polarization-related indices when CR3 was absent by adding the NOX2 activator PMA, as well as by supplementing H_2_O_2_. The results showed that the addition of PMA or H_2_O_2_ increased the mRNA expression of iNOS, TNF-α, and IL-1β in CR3-deficient microglia stimulated by LPS. It suggested that CR3 could induce M1 microglia by upregulating NOX2. Deficiency of CR3 inhibited increased exosome release from M1 microglia caused by LPS, whereas the use of H_2_O_2_ restored the LPS-induced increase in exosome release (Figure 3E). This suggests that CR3 regulates M1 microglia exosome release via modulation of NOX2 activation.

### 3.3. CR3-NOX2 Mediates M1 Microglial Exosome Release Through Syntenin-1

**Figure 4:**
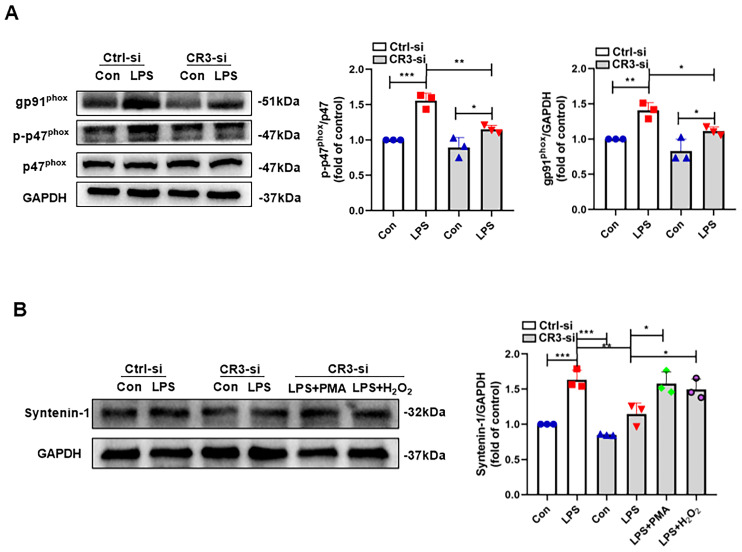
CR3-NOX2 mediates M1 microglial exosome release through syntenin-1. (**A**) Proteins p-p47^phox^, p47^phox^, and gp91^phox^ were detected using Western blot with or without CR3-si. (**B**) Protein syntenin-1 was detected using Western blot with or without CR3-si, PMA, and H_2_O_2_. * *p* < 0.05, ** *p* < 0.01, *** *p* < 0.001. n = 3.

Although we have revealed that CR3-NOX2 plays a crucial role in LPS-induced exosome release from M1 microglia, the molecular mechanism by which CR3-NOX2 regulates exosome release remains unclear. Syntenin-1 is an important regulatory molecule for exosome synthesis as well as release [18]. However, it has not been reported whether CR3-NOX2 can affect exosome synthesis by regulating this molecule. Figure 4A first clarified that CR3 regulated NOX2 activation in LPS-induced M1 microglia. We then measured the changes in syntenin-1 protein in the absence of CR3 as well as in the presence of PMA and H_2_O_2_ (Figure 4B). The results showed that syntenin-1 protein expression was significantly decreased when CR3 was deleted compared with the control group. In contrast, syntenin-1 protein expression was significantly elevated when NOX2 was activated using PMA or supplemented with H_2_O_2_ (Figure 4B). These results suggest that CR3-NOX2 regulates the synthesis and release of M1 microglia exosomes via syntenin-1.

### 3.4. CR3 Mediates M1 Microglia-Induced Neurotoxicity via NOX2

**Figure 5:**
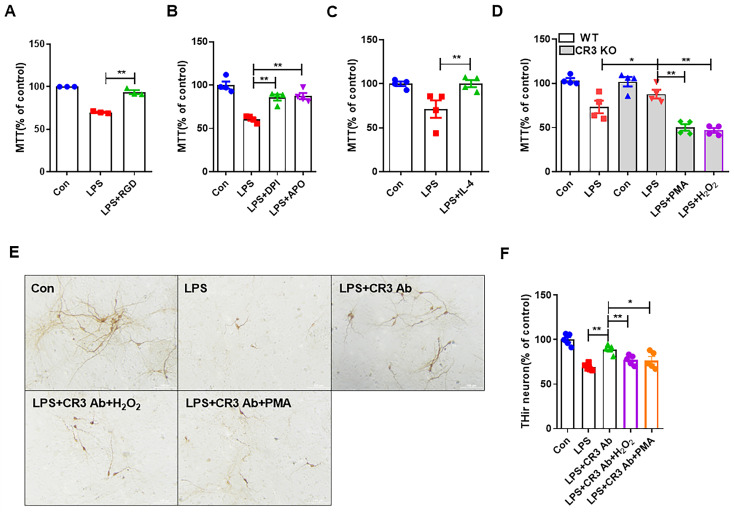
CR3 mediates M1 microglia-induced neurotoxicity via NOX2. (**A**–**C**) Supernatant was prepared from treated microglia with or without RGD, DPI, APO, and IL-4, and then transferred to SH-SY5Y cells. (**D**) Supernatant was prepared from primary midbrain neuron-glia culture extracts from WT and CR3^−/−^ mice treated with or without PMA and H_2_O_2_ and then transferred to SH-SY5Y cells. (**E**,**F**) TH immunostaining of LPS-intoxicated primary midbrain cultures with or without PMA and H_2_O_2_. Scale bar = 100 μm. * *p* < 0.05, ** *p* < 0.01. n = 3–5.

Although in the previous results we showed that LPS caused neurotoxicity by inducing the release of exosomes from M1 microglia, the role played by CR3-NOX2 in this process was not confirmed. Here, BV2 cells were treated with LPS for 24 h and administered RGD peptide (CR3 inhibitor), NADPH oxidase inhibitors (diphenyliodonium (DPI) and Apocynin (APO)), and IL-4 (M2 microglia activator). SH-SY5Y cells were then treated with BV2 cell supernatant from these groups for 24 h, and then cell survival was detected using the MTT assay. The results showed that the LPS-induced decrease in SH-SY5Y cell survival caused by M1 microglia was reversed when CR3 was inhibited, and the same results were obtained when inhibiting NOX2 or inhibiting M1 microglia (Figure 5A–C). Next, we treated primary mixed glia culture extracts from WT and CR3^−/−^ mice with LPS with or without PMA and H_2_O_2_. At the end of the treatment, the cell supernatant from each group was used to treat SH-SY5Y cells for 24 h to observe cell viability. The results in Figure 5D show that the absence of CR3 significantly alleviated the neurotoxicity induced by M1 microglia. Interestingly, the protective effects of CR3 KO were abolished by PMA and H_2_O_2_ treatment. Figure 5E demonstrates that in primary midbrain neuron-glial cultures, LPS-induced loss of dopaminergic neurons (THir cell) was alleviated after CR3 was inhibited. In the case of CR3 inhibition, neuronal damage caused by M1 microglia was recovered after supplementation of PMA and H_2_O_2_. These results confirmed the involvement of CR3-regulated NOX2 in M1 microglia-induced neurotoxicity induced by LPS.

### 3.5. CR3 Mediates Microglial M1 Polarization, NOX2 Activation, and Exosome Release in LPS-Treated Mice

**Figure 6:**
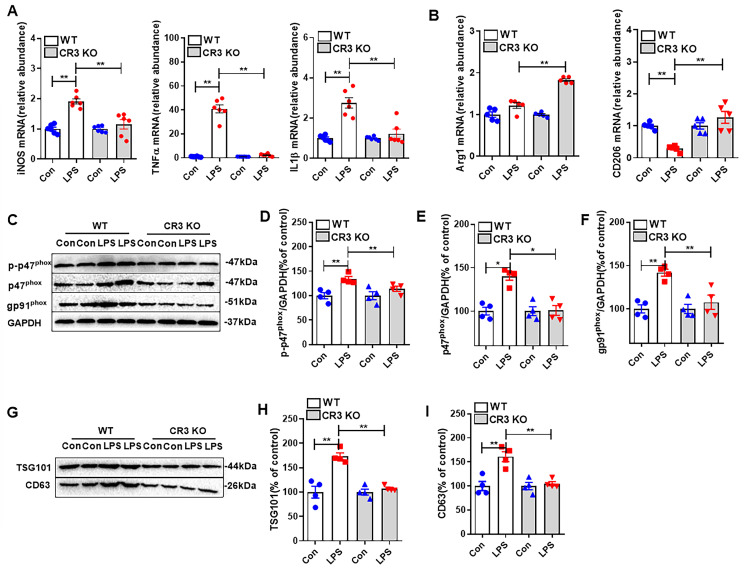
CR3 mediates microglial M1 polarization, NOX2 activation, and exosome release in the brain of LPS-treated mice. (**A**) Microglia M1 polarized mRNA (iNOS, TNF-α, and IL-1β) expression detected in the brains of WT and CR3^−/−^ mice after LPS treatment. (**B**) Microglia M2 polarized mRNA (Arg1 and CD206) expression detected in the brains of WT and CR3^−/−^ mice after LPS treatment. (**C**–**F**) The expression of NOX2 activation-related proteins p-p47^phox^, p47^phox^, and gp91^phox^ in the brains of WT and CR3^−/−^ mice after LPS treatment analyzed using Western blot. (**G**–**I**) The expression of exosome release-related proteins TSG101 and CD63 in the midbrain of WT and CR3^−/−^ mice after LPS treatment analyzed using Western blot. * *p* < 0.05, ** *p* < 0.01. n = 3–6.

We used CR3 KO mice and WT mice to observe the effects of CR3 on microglia M1 polarization, exosome release, and NOX2 activation in vivo. As shown in Figure 6A, M1 polarization-associated mRNAs (iNOS, TNF-α, and IL-1β) in the midbrain of WT mice were markedly elevated in response to LPS stimulation, whereas the expression levels of these mRNAs were significantly reduced when CR3 was knocked down. At the same time, we also examined the mRNA expression of Arg1 and CD206, markers of M2 polarization, in the midbrain of mice. The results showed that LPS failed to significantly affect the expression of Arg1 in WT mice, but when CR3 was knocked down, LPS significantly elevated the expression of Arg1. In WT mice, LPS significantly inhibited the expression of CD206, and when CR3 was knocked out, LPS significantly stimulated the expression of CD206 (Figure 6B).

Figure 6C–F demonstrate the effect of CR3 on NOX2 activation in response to LPS stimulation. We examined the expression of NOX2 activation-related proteins p-p47^phox^, p47^phox^, and gp91^phox^. The results showed that in WT mice, the expression of these proteins was significantly elevated after LPS treatment, while the expression of the three proteins was significantly reduced when CR3 was knocked down.

Next, we observed the effect of CR3 on the exosome marker. The results showed that the expression of the exosome marker proteins TSG101 and CD63 in the midbrain of WT mice treated with LPS was significantly elevated, whereas the expression of these proteins was significantly reduced when CR3 was knocked down (Figure 6G–I).

The above results confirm that the presence or absence of CR3 modulates M1 polarization, NOX2 activation, and exosome release from microglia in LPS-treated mice.

## 4. Discussion

PD is the most common form of movement disorder in older adults. Although age is an important risk factor in the pathogenesis of PD, the etiology of PD remains unclear. In recent years, the role of neuroinflammation in the development and progression of PD has become a new research topic. It is a complex network of interactions that includes both immune and non-immune cells. Microglia are resident macrophages in the CNS and play a dominant role in regulating neuroinflammation and maintaining homeostasis in the body. However, if for some reason there is a failure in effectively removing pathogens, damage, or aggregates from the brain, then microglia may become chronically activated, which may lead to neuronal damage; for example, excessive cytokine production, excessive ROS production, or the occurrence of synapses and neurons being over-phagocytized, which could lead to the development of PD [19]. There is a switch to a ‘reactive state’ in response to pathological stimuli. When M1 microglia persist, they contribute to various inflammatory diseases, including PD, by releasing pro-inflammatory factors [20].

Microglia respond to alterations in brain homeostasis by expressing chemokines, cytokines, neurotransmitters, and a variety of membrane receptors, including the integrin CD11b (CR3) [21,22]. CR3 has been documented to modulate microglia phagocytosis and migration; it can also recognize a variety of stimuli to mediate neuroinflammation and neurodegeneration [23]. In our study, the important role played by CR3 in LPS-induced M1 microglia was clarified: the presence of CR3 mediated microglia M1 polarization, whereas its effect on M2 polarization was insignificant.

The major source of oxidants in most cells is the NOX family of NADPH oxidoreductases (NOX1-5 and DUOX1-2). They regulate the production of superoxide and hydrogen peroxide through one-electron reduction of molecular oxygen [24], which is considered essential in different types of neurodegenerative diseases. Microglia are capable of expressing the highest levels of NOX2 compared to other cells in the CNS [25]. Stimulation of induced M1 microglia tended to upregulate the expression levels of NOX subunits. At the same time, NOX2-generated oxidants are paramount for the expression of a variety of pro-inflammatory factors, including (iNOS, IL-1β, TNF-α), as well as molecules associated with the M1 microglia response [26]. In microglia, NOX2 was located downstream of CR3. Upon external stimulation, the p40^phox^, p47^phox^, and p67^phox^ in the periplasm move and transfer to the cell membrane, where they bind to the p22^phox^ site and form an enzyme complex. At this time, there was a conformational shift of gp9^phox^ on the cell membrane, and electrons were transferred across the membrane, thus activating NOX2 to play its biological role. In our study, when LPS was added for stimulation, p47^phox^ in the plasma of BV2 cells moved to the cell membrane, and membrane translocation occurred. In contrast, knockdown of CR3 attenuated the movement of p47^phox^ and membrane translocation. Thus, CR3 mediated M1 microglia through activation of NOX2. Indeed, CR3 regulation of NOX2 is not a simple upstream–downstream regulation. Zhou et al. previously reported that the use of a NOX2 inhibitor, DPI, resulted in reduced CR3 expression in LPS-treated microglia [9].

Exosomes are nanoscale vesicles with a diameter of about 30–150 nm [27]. Exosomes are capable of delivering mRNA, DNA, and proteins between cells to achieve intercellular communication and regulation, and play an important role in the development of diseases, including cancer, aging, and PD [28,29,30]. Shi et al. used a labelling technique to inject labelled α-syn into the brains of mice, and found that these α-syn appeared in blood exosomes, suggesting that exosomes may play a role in PD by delivering α-syn [31]. Exosomes are not only able to deliver α-syn as ‘cargo’ but also aggregate it. It has been reported that exosomes from PD patients can enhance the accumulation of α-syn and induce neuronal deformation in PD mice in a dose-dependent manner [32,33]. An active role of microglia in the delivery of α-syn to neurons via exosomes was demonstrated by Guo et al. They found that pro-inflammatory factors released by activated microglia enhanced the aggregation and diffusion of proteins induced by the exosomes they released [34]. This result corroborates our study. In our results, LPS-induced M1 microglia caused neuronal cell damage by increasing the release of exosomes, thereby increasing the release of iNOS, TNF-α, and IL-1β. When TSG101 expression was inhibited, the release of these inflammatory factors was significantly reduced, and neuronal damage was alleviated.

Intracellular signaling and the integration and regulation of diverse signaling pathways are facilitated by multimeric protein complexes. A specific class of proteins, which lack enzymatic activity but can simultaneously bind to two or more proteins, ensures the specificity and efficiency of signaling processes. These proteins are referred to as adaptor proteins [35]. Among the adaptor proteins is a type of protein containing PDZ (postsynaptic density protein, disc large, and zonula occludens) structural domains, which are involved in a variety of important physiological and pathological activities of the organism by interacting with various proteins through the structural domains with binding activity. Syntenin is one such protein containing two PDZ structural domains, which interacts with various proteins and is involved in various important life activities such as development, cancer development, and metastasis [36,37]. The involvement of Syntenin in exosome biogenesis is a novel function discovered in recent years. Among them, syntenin-1 is the most highly expressed protein in almost all cells and in the serum of patients with PD [38], and is often used as a universal exosome marker [39]. At present, there are no reports on the role of syntenin-1 in microglial exosome release. Novelly, our results confirmed its increased expression in LPS-induced M1 microglial cells, confirming its involvement in the synthesis of microglial exosomes.

The role of Syndecan–Syntenin–ALIX in exosome biogenesis was first reported in 2012. Syndecan co-localized with syntenin-1 in the plasma membrane and intracellular vesicles and played an important role in the assembly of the cell membrane and the cytoskeleton system. The PDZ domain of Syntenin-1 promoted high-affinity interaction between syntenin-1 and syndecan. In addition, syntenin-1 directly interacted with ALIX via its LYPX(n)L motif, thereby linking syndecan to ALIX, an auxiliary component of the ESCRT machinery that supports endosomal membrane budding and shedding [40,41]. The results of Qiao et al. confirmed that tetraspanins (TSPAN6) interact with the N terminus of syntenin-1, PDZ1 domain, and PDZ2 domain through their C terminus, then regulate the secretion of adipose-derived stem cells-derived exosomes (ADSCs-Exos) [42]. Our results show that CR3 regulates LPS-induced exosome synthesis and secretion in M1 microglia via NOX2. However, the mechanism by which syntenin-1 was recruited and combined with CR3-NOX2 is unclear and will be addressed in our next experiments. It has been reported that the PDZ domain is mainly composed of 80 to 90 amino acid residues, forming a globular structure that is mainly composed of 6 β-fold chains and two α-helix chains. The C-terminal peptide of the binding protein can insert into the groove between the β2 chain and the α2 helix to interact with it [43,44]. Currently, we hypothesize that CR3-NOX2 can regulate the binding of an unknown protein to the N-terminus of syntenin-1 or to an institutional domain of the PDZ to mediate the synthesis and secretion of syntenin-1 and thus the regulation of exosomes in M1 microglia.

In summary, we found that microglia exosome release is dependent on the regulation of CR3. The CR3-NOX2 axis promoted microglia M1 polarization and exosome release through the modulation of syntenin-1, and caused toxicity to neurons. Our findings highlight the important role of the CR3-NOX2 axis in microglia M1 polarization-induced inflammation and neurotoxicity and provide novel mechanisms. However, due to the limitations of the experimental equipment and conditions, our quantitative study on the relationship between the content of microglia exosome release and its response to neuronal injury is deficient, and even though we were able to confirm that microglia M1 polarization can damage neurons through exosome release, the specific molecular mechanism and the extent of the damage need to be further investigated. However, this study does provide a new perspective and part of the evidence for the next research direction.

## 5. Conclusions

Overall, our findings revealed a critical role of the CR3-NOX2 axis in controlling microglial exosome release and related neurotoxicity through syntenin-1. We first demonstrated that CR3 regulates microglia M1 polarization and exosome release. It was further demonstrated that CR3 exerts this role by regulating NOX2. Finally, we revealed that the CR3-NOX2 axis can promote exosome release and cause neuroinflammation and neuronal toxicity through the regulation of syntenin-1, providing a novel target for the development of a therapeutic strategy for neuroinflammation-mediated neurodegeneration. 

## Data Availability

The data that support the findings of this study are available from the corresponding author upon reasonable request.
